# Noninvasive and Targeted Gene Delivery into the Brain Using Microbubble-Facilitated Focused Ultrasound

**DOI:** 10.1371/journal.pone.0057682

**Published:** 2013-02-27

**Authors:** Po-Hung Hsu, Kuo-Chen Wei, Chiung-Yin Huang, Chih-Jen Wen, Tzu-Chen Yen, Chao-Lin Liu, Ya-Tin Lin, Jin-Chung Chen, Chia-Rui Shen, Hao-Li Liu

**Affiliations:** 1 Department of Electrical Engineering, Chang-Gung University, Taoyuan, Taiwan; 2 Department of Neurosurgery, Chang-Gung University and Memorial Hospital, Taoyuan, Taiwan; 3 Molecular Imaging Center, Chang-Gung University and Memorial Hospital, Taoyuan, Taiwan; 4 Department of Nuclear Medicine, Chang-Gung University and Memorial Hospital, Taoyuan, Taiwan; 5 Department of Chemical Engineering, Min-Chi University of Technology, Taipei, Taiwan; 6 Graduate Institute of Biomedical Sciences, Chang-Gung University, Taoyuan, Taiwan; 7 Department of Medical Biotechnology and Laboratory Science, Medical College, Chang-Gung University, Taoyuan, Taiwan; University of Chicago, United States of America

## Abstract

Recombinant adeno-associated viral (rAAV) vectors are potentially powerful tools for gene therapy of CNS diseases, but their penetration into brain parenchyma is severely limited by the blood-brain barrier (BBB) and current delivery relies on invasive stereotactic injection. Here we evaluate the local, targeted delivery of rAAV vectors into the brains of mice by noninvasive, reversible, microbubble-facilitated focused ultrasound (FUS), resulting in BBB opening that can be monitored and controlled by magnetic resonance imaging (MRI). Using this method, we found that IV-administered AAV2-GFP (green fluorescence protein) with a low viral vector titer (1×10^9^ vg/g) can successfully penetrate the BBB-opened brain regions to express GFP. We show that MRI monitoring of BBB-opening could serve as an indicator of the scale and distribution of AAV transduction. Transduction peaked at 3 weeks and neurons and astrocytes were affected. This novel, noninvasive delivery approach could significantly broaden the application of AAV-viral-vector-based genes for treatment of CNS diseases.

## Introduction

Gene therapy is a potentially powerful means of treatment of various diseases with genomic causes. Recombinant adeno-associated viral (rAAV) vectors provide several advantages including no pathogenicity, typical persistence of the transgene as an episome, low immunogenicity, complete removal of all viral genes, and long-term gene expression [1]. AAV serotype 2 (AAV2) vectors have been most intensively studied for the treatment of various diseases, and in clinical trials for Canavan's [2], Batten's [3], Parkinson's [4], and Alzheimer's diseases [5]. Such central nervous system (CNS) disorders are important targets for gene therapy, but the delivery of therapeutic proteins and or genes to the brain presents a major challenge. Current attempts to deliver AAV vectors for the treatment of CNS diseases rely on local, direct injection into the brain [4], [5], but the region of recombinant gene-expression, is severely limited mainly due to the existence of the blood-brain barrier (BBB).

Intravenous (IV) administration of viral vector delivery is comparable less invasive, however, it appears to be ineffective for CNS delivery due to the BBB blockage. The BBB is formed by tight junctions between the endothelial cells of the cerebral capillaries and blocks AAV diffusion and entrance from the blood stream to the brain parenchyma [6]. Recent human trials using rAAV-2 vectors all involved treatment of neurological disorders within large brain regions such as cortex, and found limited distribution of rAAV-2 after intracranial injection [1], [4], [7]. Direct gene-carrying AAV injection into the brain parenchyma allows transfection that can circumvent the BBB obstruction; however, the transfected cells only limited to the CNS tissues surrounding the tract and cannot be widely spread. Convection-enhanced delivery (CED) that infuses macromolecules or AAVs actively has been proposed to increase the distance of penetration after viral vector direct injection [8]. However, these procedures are invasive and subject to additional risks associated with surgery since Burr holes are required for insertion of the infusion tube [9], [10]. The BBB can be globally disrupted by intra-arterial infusion of osmotic agents such as mannitol to increase BBB permeability has been attempted [11], [12], and the concept of combining osmotic BBB-opening with viral vectors to achieve CNS gene-expression was proposed [13]. However, osmotic infusion creates systemic BBB opening and specific targeted gene expression cannot be controlled. Recently, the use of self-complementary AAV serotypes 9 (scAAV9) vector through intravenous injection can infect CNS cells [14], [15]. However, with this viral vector dose (reaching 10^12^ vg), organs such as liver and heart would be significantly infected and it remains viral toxicity concerns.

Microbubble-enhanced focused ultrasound (FUS) has been shown to locally and temporally disrupt the BBB [16]–[18]. FUS energy is capable of transcranial penetration [19]–[21], thus providing an entirely noninvasive method for local and transient disruption of the BBB. Moreover, contrast-enhanced magnetic resonance imaging (CE-MRI) can be used to observe, monitor and guide the distribution of BBB-opened regions [16], [17]. FUS-induced BBB opening can be exploited for the local delivery of small anti-cancer chemotherapeutic drugs [22], [23], large therapeutic antibodies [19], or therapeutic macromolecules [24], [25]. Recently, FUS BBB-opening with the presence of a specially designed plasmid-conjugated microbubbles already demonstrated the possibility of local CNS gene expression [26]. We reasoned that the high therapeutic potential of viral vectors could potentially be realized by replacing traditional direct viral injection into the brain with noninvasive, transcranial FUS delivery. Here we demonstrate for the first time the feasibility and stability of delivering AAV vectors into the brain for gene expression, using IV administration of AAV combined with noninvasive focused-ultrasound in mice. We show that gene transduction in the BBB-opened brain can be successfully and stably produced with a low viral titer of 1×10^9^ viral genomes.

## Materials and Methods

### Experimental design

All animal experiments were approved by the Institutional Animal Care and Use Committee of Chang Gung University and adhered to their experimental animal care guidelines (IACUC approval number: CGU08-58). Animal were shaved and a PE-10 catheter was inserted in the tail vein for substance administration during the experiment. Seventy-four outbred imprinting-control-region (ICR) mice (male; 7–8 weeks old) were separated into two groups. Group 1 consisted of twenty-nine mice that were used to optimize the time course of AAV infection from 1 to 6 weeks (Table S1). Group 2 consisted of forty-five mice that were used to evaluate the efficiency of our approach at the optimized time for infection of 2–3 weeks.

### Preparation of the recombinant adeno-associated virus (rAAV)

Recombinant adeno-associated virus (AAV) is a proven research and therapeutic tool [27], and commercial AAV Helper-Free system (Stratagene, La Jolla, CA) were employed. The vector system contains the necessary genes from adenovirus (pHelper vector) to induce the lytic phase of AAV producing recombinant, replication-defective AAV virions ready to deliver a gene of interest to target cells. The pAAV-hrGFP is replication-deficient AAV vector, which encodes the Renilla reniformis green fluorescent protein (hrGFP) gene by the CMV promoter. Standard AAV-hrGFP virus production was performed and was described in detail previously [28]. Briefly, plasmid DNA pAAV-hrGFP plus the pRC vector encoding Rep and Cap proteins and the pHelper vector encoding the adenovirus gene products was used to transfect 293T cells at an 80% confluence stage. The cell lysates were collected 48 hours post-transfection and purified by CsCl density gradient centrifugation. The titers of pAAV-hrGFP were determined using real-time RT-PCR analysis by calculating the viral genome copy number.

### Cloning and generation of recombinant adeno-associated virus

Virus containing pAAV2-IRES-hrGFP was produced with the AAV2 helper system (Stratagene, La Jolla, CA). Briefly, plasmid DNA (pAAV2-IRES-hrGFP plasmid plus the pRC vector encoding Rep and Cap proteins and the pHelper vector encoding adenoviral gene products) was used to transfect 293T cells at 80% confluence. Cell lysates were collected 48 hr post-transfection and purified by CsCl density gradient centrifugation. Titers of rAAV-hrGFP were determined by RT-PCR analysis by calculating the viral genome copy number. The biodistribution of the employed pAAV2-IRES-hrGFP transduction were mainly found in liver and spleen [29]–[31], and can have high transduction efficiency in other organs when specific promoters were designed [28]; the neurotoxicity were not detected when the viral particle titer below than 10^11^ vg (viral genomes) with the inject volume of 30 µL in mice [32], [33].

### Focused ultrasound and viral vector delivery

Animals were anesthetized with a mixture of oxygen (flow rate: 0.8 L/min) and 2% vaporized isoflurane using an anesthesia vaporizer. The top of the cranium was shaved with clippers, and a PE-10 catheter was inserted into the tail vein for injections. The animal was placed directly under an acrylic water tank with its head attached tightly to the thin-film, 4×4 cm^2^ window at the bottom of the tank. 30 µL of viral vectors with the titer of 1×10^9^ viral genomes per gram (vg/g) of treated animal were bolus injected through the same PE-10 catheter and immediately followed a 0.3 mL/kg microbubbles bolus injection (SonoVue^®^ SF6-coated ultrasound microbubbles, mean diameter 2–5 µm, 2.5 µg/kg, Bracco Diagnostics Inc., Milan, Italy; 0.03 mL/kg for clinical diagnostic application) mixed with 20 µL of saline. The center of the focal zone was placed at a 2–3 mm depth of penetration for each hemisphere. With a 10-second delay, ultrasonic energy was delivered to the brain transcranially using a spherically focused transducer (Imasonics, Besancon, France; diameter = 60 mm, radius of curvature = 80 mm, frequency = 1.5 MHz; negative peak pressure when considering mouse-skull insert loss was measured; the measured half-maximum pressure amplitude diameter and length of the produced focal spot were 2 and 10 mm, respectively; detailed FUS field calibration please see the Supporting Information). Burst-mode ultrasound with burst length 10 ms, pulse-repetition frequency (PRF) 1 Hz, and duration 120 seconds was used. The input electric power was 3–8 W (monitored by a RF-power meter (Bird model 4421, USA); corresponding acoustic pressure amplitude of 0.44–0.7 MPa). Animals were sacrificed 1–6 weeks later. The recovery process from ultrasound-induced brain damage was carefully monitored. Besides of the FUS experimental group, three control experiments were performed: (1) FUS only without AAV delivery (n = 2; 0.7-MPa; 3 weeks after AAV transduction); (2) viral vector IV administration only without FUS exposure (n = 2; 3 weeks after AAV transduction); (3) direct viral vector injection (same AAV-2 viral titer but with a reduced volume of 3 µL suggested by previous report [34]) with a micro-injection pump system as positive control (n = 5; 3 weeks after AAV transduction).

### Magnetic resonance imaging (MRI)

The degree of BBB opening was monitored with a 7-Tesla magnetic resonance scanner (Bruker ClinScan, Germany) and a 4-channel surface coil was used on the top of the mouse brain. A gradient echo FLASH sequence was performed to acquire T1W images (pulse repetition time (TR)/echo time (TE) = 300/3.81 msec; FOV = 21×25 mm^2^; in-plane resolution = 173×256 mm^2^; slice thickness = 0.5 mm; flip angle = 70°; acquisition time = 156 seconds).

### Immunofluorescence (IF)

Animals were sacrificed 2–3 weeks after FUS sonication. The brains of these mice were quickly removed and frozen for embedding in Optimal Compound Temperature compound (Tissue-Tek O.C.T. Compound, Sakura Finetek). Embedded brains were sectioned serially into 10-μm-thick slices with a cryostat microtome (CM3050S, Leica, Germany). After washing with PBS, slides were subjected to immunoflurorescence (IF) with a fluorescent microscope (TissueFAX Plus, TissueGnostics, Austria) to confirm the expression and determine the distribution of AAV2-GFP protein in the brain. Sections were fixed with 4% paraformaldehyde, rinsed with PBS, blocked with serum (10% normal goat serum, S-1000, Vector laboratories, CA, USA) for 30 min at room temperature, and incubated with rabbit-anti-GFP polyclonal antibody (1∶200, Covalab, Cambridge, UK) at 4 °C overnight. After rinsing with PBS, the sections were incubated with goat-anti-rabbit IgG conjugated with rhodamine (1∶100, Millipore, Billerica, MA, USA) for 1 hour at room temperature in the dark. To distinguish the AAV-transfected cell type, mouse-anti-neuronal nuclei (NeuN) monoclonal antibody (1∶100, Millipore, Billerica, MA, USA) and rabbit-anti-Glial Fibrillary Acidic Protein (GFAP) polyclonal antibody (1∶100, Dako, Glostrup, Denmark) were then used on the same sections, then incubated with Cy3-conjugated Goat-anti-mouse (1∶200, Jackson ImmunoResearch, 115-165-003) and Cy3-conjugated Goat-anti-rabbit (1∶200, Jackson ImmunoResearch, 111-165-003) secondary antibodies respectively. Images were acquired and merged, to determine which cell types has been transfected. Finally, every section was stained with hematoxylin and eosin (HE) for histological examination.

### Correlation between MR-predicted and optically-detected AAV infection

Animals (n = 26) were well positioned in the MR bore (within 1-minute time lapse after FUS exposure) with the animal head attached with a receive-only surface loop coil (diameter 20 mm). Animals were anesthetized with isoflurane (1.5–2% with 0.6L /min O_2_ flow rate). T1-weighted MR images were acquired immediately after the completion of Gd-DTPA (0.25 mL/kg, Magnevist^®^; Berlex Laboratories, Wayne, NJ) administration followed by flushing with saline (0.5 mL/kg) and heparin (0.2 mL/kg) to identify the BBB-disrupted region (with 10-second time lapse before acquiring MRI). In contrast, AAV2-GFP expression and distribution in tissue was detected by fluorescence microscopy, so these two modalities could be combined to predict the efficiency of AAV infection.

### Quantitative analysis of GFP-positive area and statistical analysis

Quantitative analysis of the GFP-positive area was performed on a personal computer running image processing software (Image J^®^, Matlab^®^) interfaced with a digital CCD camera mounted on an fluorescence microscope (TissueFAX Plus, TissueGnostics, Austria). Results are expressed as means ± standard error (SE) of duplicate or more measurements, obtained from three or more independent experiments. Data were analyzed using one-way ANOVA followed by un-paired post-ANOVA tests using the Bonferroni correction. Values of p<0.05 were considered statistically significant. Cell transduction rate was calculated by counting the ratio of GFP-positive cell among the total cell number per selected region-of-interest (100×100 µm^2^) in each IHC stained slide (n = 12; animals sacrificed at week 2 or 3).

## Results

### CE-MRI reveals FUS-induced BBB opened regions and serves as an indicator of AAV2 vector delivery and GFP expression

**Figure 1:**
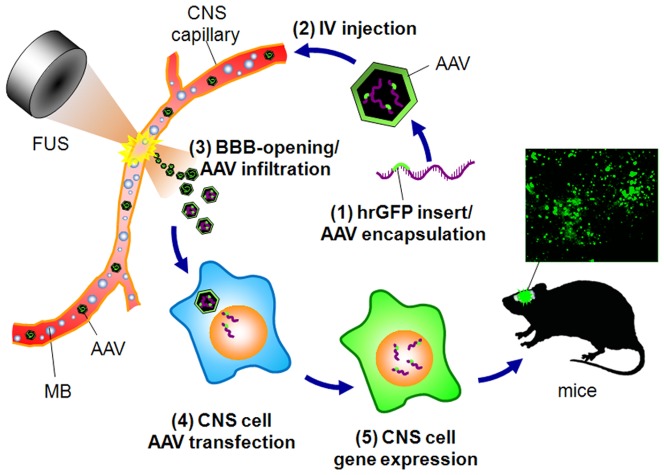
Scheme of the study. AAV2 harboring a specific gene (hrGFP) is IV-injected into animals. Delivery of focused ultrasound to the animal brain causes virus to leak into the brain through the opened BBB, where it transfects cells. Transfected cells will produce cytoplasmic GFP that can be detected by immunofluoresence microscopy of mouse brains.

**Figure 2:**
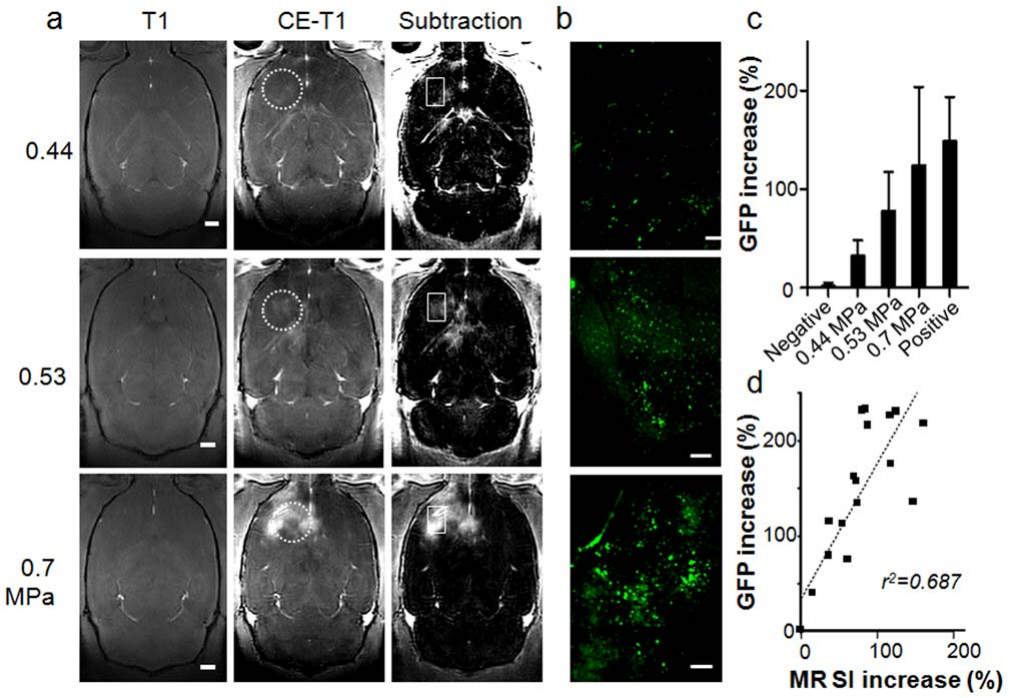
Correlation between MRI and GFP expressions. (a) T1-weighted MR images showing BBB disruption by different acoustic pressures (bar = 1 mm) and (b) respective GFP expression by immunofluorescence (bar = 200 µm). (c) Increase in GFP signal intensity at different acoustic powers. (d) Correlation of the change in MR signal intensity with the increase in AAV2-GFP expression (r^2^ = 0.687). In (a), circles in dashed lines represent the FUS exposure area, and the rectangular regions represents the zoomed immunofluorescence regions.

Our experimental procedure is summarized in Fig. 1. First, we verified the use of MRI to monitor FUS-induced BBB opening. Typical CE-MRI results and AAV2-GFP expression for three different acoustic powers are shown in Fig. 2 (3 weeks after AAV transduction; n = 26). Comparison of MR images before and after Gd-DTPA administration did not show any difference between the two brain hemispheres in sham control animals. Low power (0.44 MPa) sonication induced modest BBB opening indicated by a slight change in signal intensity (SI) (26%), whereas 0.7-MPa sonication induced a more profound BBB-opening effect with more apparent contrast agent leakage (92%) (Fig. 2a). These local BBB-opening effects were confirmed as EB staining in experimental but not contralateral brain hemispheres (Fig. S1) and by increased EB dye extravasation at higher acoustic power (Fig. S2) (EB concentration increase of 97.7%, 508.2% and 726.5% were measured in 0.44-, 0.53-, and 0.7-MPa exposure, respectively).

When brain sections were observed 3 weeks later, the GFP expression sites colocalized well with the contrast-enhanced regions observed in T1-MR images from the same animals (Fig. 2b). Low power 0.44-MPa sonication produced stable and significant GFP expression compared to the contralateral brain (32.3%, p<0.05), whereas 0.7-MPa sonication resulted in higher local GFP expression (124.3%, p<0.05) compared to contralateral brain (Fig. 2c). No GFP expression could be detected in FUS-only or AAV-2-only animals. Transmission electron micrographs (TEMs) demonstrated that 0.53-/0.7-MPa FUS-mediated brains produced caveolae and cytoplasmic vacuolar structures similar to previously reported observations [35], that may promote widening of intra-endothelial tight-junction crafts to facilitate the passage of more AAV2 vectors (Fig. S3). 0.7-MPa FUS exposures typically induced relatively larger inter-endotelial tight-junction crafts on capillaries than the 0.53-MPa cases.

We observed a high correlation between GFP expression and MRI signal increase (r^2^ = 0.687; Fig. 2d), indicating that a higher degree of BBB-opening induced a greater level of AAV transfection and expression. The signal enhancement from MRI (obtained immediately after FUS exposure) could thus potentially serve as a useful indicator of AAV-encapsulated gene delivery and expression (as observed 3 weeks after FUS exposure in our experiments).

### Optimization of GFP expression facilitated by FUS-induced BBB-opening

**Figure 3:**
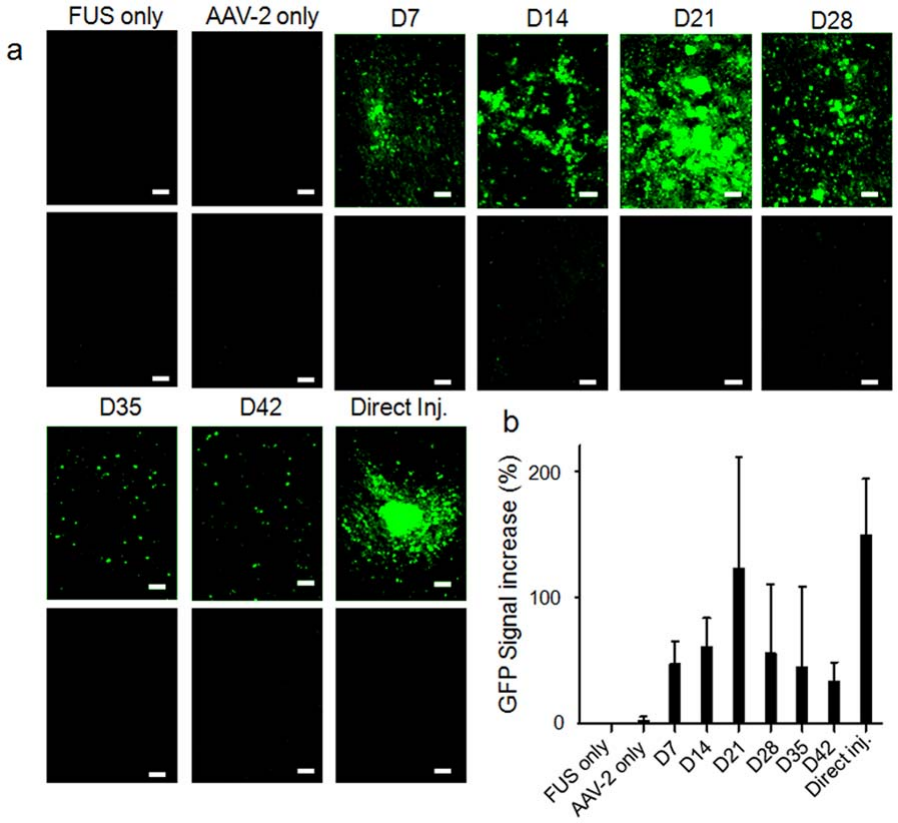
Longitudinal GFP-expression profiles. (a) GFP expression assayed weekly from week 1 (D7) to week 6 (D42), and in negative and positive controls. Top panels, sonicated brain; lower panels, contralateral control. Bar = 100 µm. (b) Evaluation of increase in GFP signal intensity at different times after transfection.

Next we analyzed the AAV2-GFP transduction period to determine the kinetics of infection in the BBB-opened brain region (Fig. 3a; control and 0.7-MPa FUS-mediated animals; n = 39). In the absence of BBB-opening, we did not detect any GFP expression in either brain hemisphere (negative control; sacrificed at week 3). GFP expression could be observed within the first week after delivery of 0.7-MPa FUS and reached a maximum level at week 3 (122.8%), after which it decayed. GFP remained detectable at week 6 (33.5%). GFP expression in this positive control brain was locally concentrated at the injection tract with limited distant diffusion. Thus injected AAV was only capable of transducing cells located in the vicinity of the BBB-breakdown injection track region but could not infect the parenchyma containing an intact BBB structure. The GFP expression level from direct AAV injection was similar to that observed at the peak time point (week 3) of the FUS approach (148.7% vs. 122.8%), indicating that FUS provided equivalent AAV gene transfection efficacy compared to direct cranial AAV injection (Fig. 3b).

**Figure 4:**
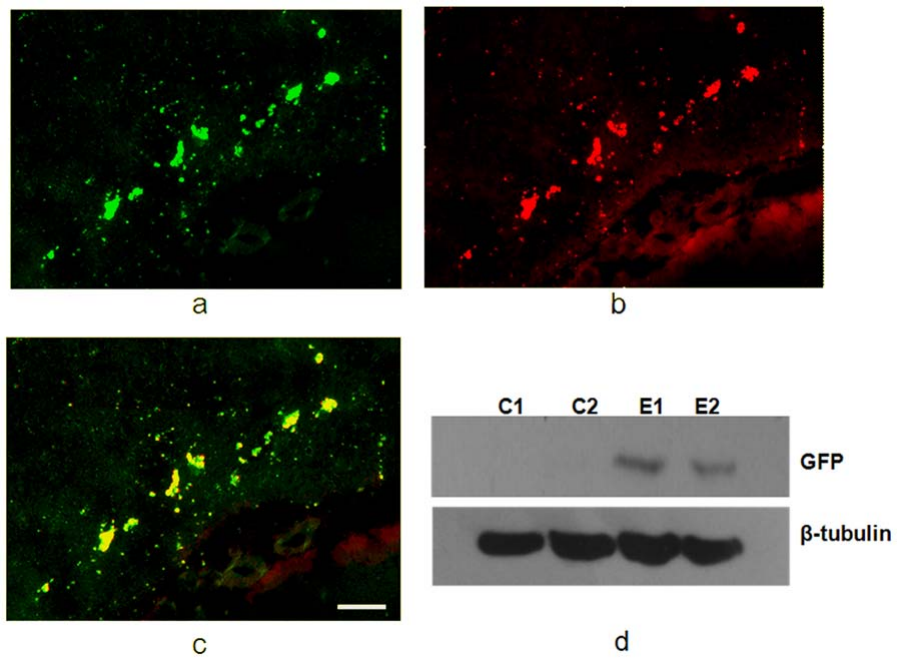
Confirmation of GFP expression by immunofluorescence and Western blot (3 weeks after AAV transduction). (a) AAV2-GFP expression as detected by immunofluorescence of GFP; (b) anti-GFP rhodamine immunofluorescence; (c) overlaid GFP expression and anti-GFP rhodamine to demonstrate their colocalization; (d) Western blot of β-tubulin and GFP expression in control and FUS-exposed brains, demonstrating that GFP was only expressed in the experimental group. Bar = 100 µm.

Colocalization of the GFP fluorescence signal and anti-GFP rhodamine immunofluorescence (Fig. 4a-c) confirmed that the fluorescence originated and GFP expression transducted from the AAV2-GFP vector (3 weeks after AAV transduction). Western blotting also confirmed robust expression of GFP in the experimental but not in the control animals (n = 2 per each) in the FUS BBB-opened region (Fig. 4d).

### AAV2-GFP vector delivery through FUS tends to transduce GFP expression in astrocytes

**Figure 5:**
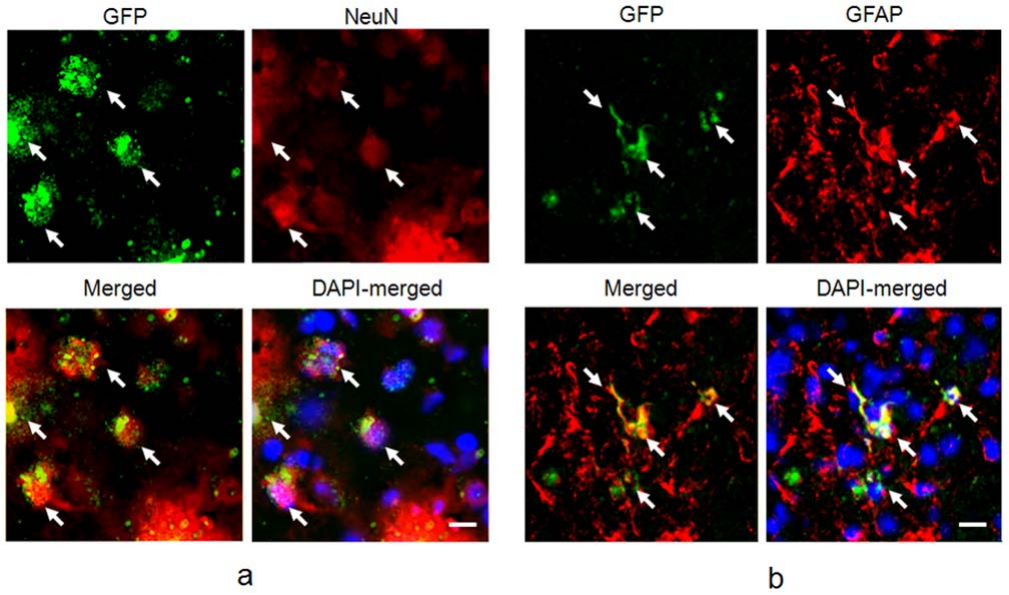
Colocalization of AAV-infected area with other markers (3 weeks after AAV transduction). (a) GFP (green) and neuronal nuclei (NeuN, red) staining, bar = 10 µm.; (b) GFP (green) and GFAP (red) colocalization by fluorescence microscopy, bar = 10 µm.

To confirm the cell type being transduced by FUS-mediated delivery of AAV2-GFP vectors, we analyzed colocalization of GFP expression with immunofluorescent staining by anti-Glial Fibrillary Acidic Protein (GFAP) and neuronal marker NeuN (n = 18; 3 weeks after AAV transduction). GFP signal from AAV2-GFP clearly colocalized with GFAP, demonstrating that the viral vectors successfully transduced astrocytes (Fig. 5a). A small amount of NeuN/GFP colocalization was also observed, indicating that transduction may also have occurred in neurons, although in a minority of cases (Fig. 5b). The AAV transduction rate in astrocytes and neurons achieved 40.88% and 12.04% in the FUS-BBB opening brain regions respectively, whereas the transduction rates in contralateral brains were 2.81% and 0%, respectively (Fig. S4). We also observed a marked recruitment of astrocytes, whereas the number of neurons remained the same in the exposed brain compared to the contralateral control (Fig. S5). These data indicated infiltration of astrocytes, without neuronal cell loss, upon AAV transfection. HE staining of the adjacent brain slice demonstrated that the regions that had undergone FUS exposure and GFP-expression were intact without any apparent tissue damage (Fig. S5).

## Discussion

In this study, we demonstrated the use of microbubble-facilitated FUS to enhance local BBB opening that successfully facilitated local AAV-2 penetration into the brain parenchyma and produced gene expression in CNS cells in mice. Unlike current invasive procedures involving direct local injection of viral vector, our FUS procedure concentrated ultrasound transcranially to induce local BBB opening for expressing genes at specific target regions in a noninvasive manner. We also demonstrated that GFP expression correlated well with MRI signal enhancement, suggesting the possibility of using MRI-monitored BBB-opening can not only served as an indicator of therapeutic agent amount [19], [36], but also can be used as the indicator of scale and distribution of AAV-transduction. It was found that FUS-mediated and direct injection provided equivalent levels of GFP expression when a same viral vector titer of was administered (1×10^9^ vg/g). This suggests the combined use of viral-vector intravenous administration with FUS-BBB opening is a potential technique to achieve targeted gene delivery for CNS disease treatment noninvasively.

Previous preclinical small-animal studies have shown that the viral particle concentration equivalent or higher than this study do not cause immune related concerns [2], and clinical trial as well as a number of preclinical primate studies already confirmed the AAV viral vector safety with the titer reaching 10^12^ vg/g [4], [37]–[39]. The selected viral vector titer of 10^9^ vg/g employed in this study is relatively low and nearly the currently reported lowest dose to perform AAV gene delivery (at least 10^10^ vg/g was administered IV to perform scAAV9 CNS gene delivery reported previously [14], [15]). Therefore, the given viral vector in this study should be of no safety concern, and there should be highly possible to further improve the gene expression rate and transduction distribution when higher titer of viral vector is employed.

It is well documented for rAAV vectors based mostly on AAV-2 for gene therapy. Although AAV-2-based rAAV vectors can transduce muscle, liver, brain, retina, and lungs, it requires several days to weeks for optimal expression. Maximum CNS transduction of AAV-2 in CNS cells through the proposed approach was observed to be 2–3 weeks. For other cell types, Ryals et al. found that an increase in gene product was clearly seen from 3 days post infection to ARPE19 cells, and maximal AAV-mediated transgene expression was reached between 7 and 14 days post infection [40]. Similarly, Sarra et al. examined the temporal and spatial pattern of GFP expression following sub-retinal injection of rAAV in the mouse and some transgene expression was reported as early as three days after injection [41]. However, the photoreceptor transduction rate increased constantly over time with efficiencies of around 15% at seven days, 40% at 14 days and reaching 70% at 120 days of assessment. Longer assessment intervals (180 and 365 days) revealed that GFP expression remained stable at high levels around 90% [41]. In contrast, although there appeared to be a strong and sustained RPE-gene transduction, transduced RPE-gene were not detected until after one week [41]. Such observation was considered due to the variety of cell types and their specific host factors. Indeed, an earlier study of applying vector administration in the monkey demonstrated that the apparent inability of rAAV to transduce cones [42] might be explained by the lack of the appropriate surface receptors for AAV on cones or due to the inability of the CMV promotor to function in such cell type.

Viral gene delivery is critically dependent on matching the right vector to the desired cell type that needs to be transduced. There have been numerous reports of successful astrocytes transduction using various viral vectors. These include a report of in vitro astrocytes transduction by AAV packaged with a specific promoter [43], transduction of astrocytes and oligodendrocytes by AAV or AAV serotypes 8 and 9 when driven by cell-specific promoters (GFAP and myelin basic protein (MBP)) [34], [44], and an intense GFP signal observed in astrocytes located in the corpus callosum using AAV-2 [45].

Since viral transduction of specific cells relies on the promoters that drive viral vectors, it is important to select promoters that will result in efficient gene expression in the selected target cells. Earlier studies have characterized the specific cell types transduced after parenchymal injection of rAAV2 encoding β-galactosidase under control of the CMV immediate early enhancer/promoter to be predominantly neurons, with an occasional transgene-expressing astrocytes [46], [47]. In contrast, the intrastriatal injection of rAAV2 transduced predominantly parenchymal cells [48]. Here we found the major transgene-expressing cells were astrocytes after FUS treatment. It is known that rAAV2 transduction depends on cell surface heparan sulfate proteoglycans (HPSGs) [49], which have multiple functions relevant to the control of the CNS injury response [50] involving the reactive astrocytes. There appears to be no doubt that these HPSG expressing cells could be transduced by rAAV2.

In this study we showed transduction of astrocytes by AAV2-GFP delivered through FUS-BBB opening. Astrocytes have traditionally been considered to be merely neuronal supporter cells, but recent studies demonstrated that they also contribute to the pathogenesis of neurodegenerative disorders[51], [52]. To date, attempts to target transgenes to glial cell populations by varying cellular promoters have resulted in limited transduction only [52], [53]. Since most reports of AAV viral-vector CNS delivery have been of neuron transduction [11], [54], [55], AAV2-GFP may be a useful tool for efficient astrocytes transduction to treat neurodegenerative diseases.

## Supporting Information

Figure S1 **EB-stained brain sections after FUS-BBB opening.** Coronal sections to display the distribution of BBB disruption by EB staining in the focal region of left brain for three different acoustic pressures: 0.44 MPa, 0.53 MPa and 0.7 MPa. Right brain: no FUS sonication. (Bar: 5 mm; C: cortex; S: striatum).
(TIF)

Figure S2 **Quantitative analysis of EB extravasation.** (a) Calibration curve of known EB standards and OD values. (b) EB quantities determined from the standard curve for three different acoustic powers. Results are indicated as means and SEM values for the experimental and contralateral brains; n = 3. (c) Percent increase in EB compared to control for three different acoustic powers. 97.65%, 508.2% and 726.49%, respectively of the EB leakage increase was observed in 0.44-, 0.53- and 0.7-MPa sonicated brains.
(TIF)

Figure S3 **Transmission electron micrographs (TEMs) of control and FUS-exposed CNS capillaries.** (a) Control capillary showing intact tight junction structure (bar = 115 nm). (b) Capillaries after 0.53-MPa FUS exposure revealing compromised tight junctions and numerous vesicles in endothelial cell cytoplasm (bar = 115 nm); (c) magnified tight junction from (b) showing inter-endothelial craft induced by FUS (bar = 1500 nm); (d) capillaries with 0.7-MPa FUS exposure showing large inter-endothelial tight-junction craft (bar = 115 nm).
(TIF)

Figure S4 **Cell-type specific AAV transduction.** Here we show the comparison of AAV transduction rate in glial cells and neurons in control and FUS-BBB opened brain regions.
(TIF)

Figure S5 **Immunofluorescence confirmation of AAV2-GFP expression.** Neuronal Nuclei (NeuN) and Glial Fibrillary Acidic Protein (GFAP) immunofluorescence, and HE staining in (a) contralateral and (b) experimental brain. Neurons (nuclei stained by NeuN) appeared similar between the two sides of the brain, but glial cells were increased in the experimental lateral brain. HE-staining showed that the tissue structure was not severely damaged by FUS treatment. Bar = 200 µm.
(TIF)

Table S1 **Summary of numbers of animal used for focused ultrasound experiments.**
(DOCX)

Method S1 **Cloning of AMCase and setup of AMCase-overexpressing cell line and rAAV.** This supplemental methods section provides a detailed description of the AMCase cloning, the setup of AMCase-overexpressing cell line and the production of recombinant AAV.
(DOCX)

Method S2 **Real-time PCR and Western blotting.** This supplemental methods section provides a detailed description of the use of real-time PCR and Western blotting to confirm the GFP expression in the brain.
(DOCX)

Method S3 **Focused ultrasound calibration and assessment of blood-brain barrier disruption.** This supplemental methods section provides a detailed description of focused ultrasound calibration and measurement, as well as the use of Evans Blue (EB) infiltration and staining to assess the BBB-opening.
(DOCX)

Method S4 **AAV direct injection as a positive control.** This supplemental methods section provides a detailed description of the AAV direct injection as a positive control group.
(DOCX)
