# Supplementary figures and images for: Noninvasive and Targeted Gene Delivery into the Brain Using Microbubble-Facilitated Focused Ultrasound
Source: PLoS One. 2013 Feb 27;8(2):e57682. doi: 10.1371/journal.pone.0057682 (PMC3584045; doi:10.1371/journal.pone.0057682)

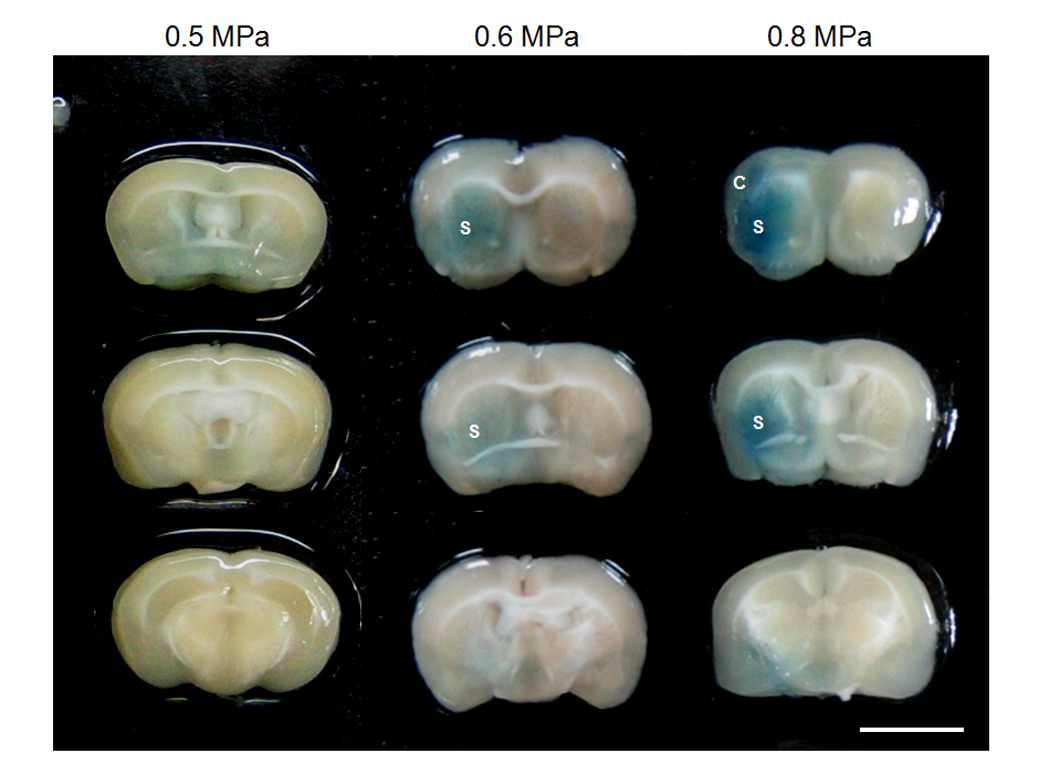

Supplement: Figure S1 — EB-stained brain sections after FUS-BBB opening. Coronal sections to display the distribution of BBB disruption by EB staining in the focal region of left brain for three different acoustic pressures: 0.44 MPa, 0.53 MPa and 0.7 MPa. Right brain: no FUS sonication. (Bar: 5 mm; C: cortex; S: striatum). (TIF) [file pone.0057682.s001.tif]

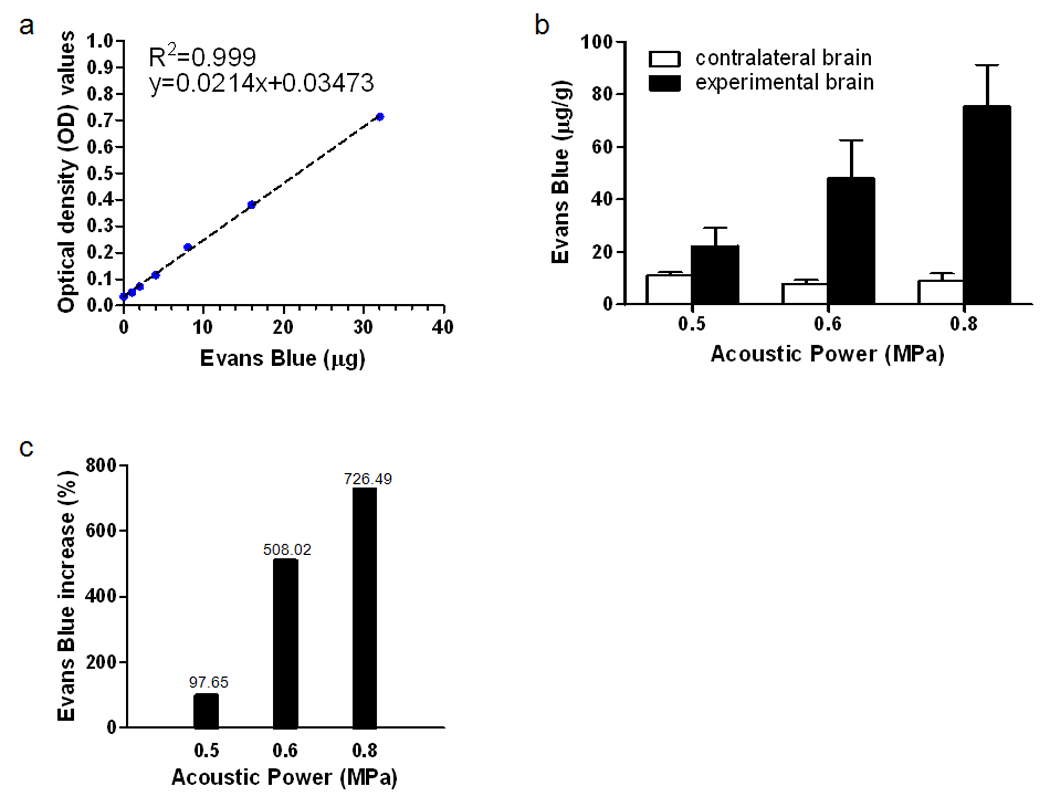

Supplement: Figure S2 — Quantitative analysis of EB extravasation. (a) Calibration curve of known EB standards and OD values. (b) EB quantities determined from the standard curve for three different acoustic powers. Results are indicated as means and SEM values for the experimental and contralateral brains; n = 3. (c) Percent increase in EB compared to control for three different acoustic powers. 97.65%, 508.2% and 726.49%, respectively of the EB leakage increase was observed in 0.44-, 0.53- and 0.7-MPa sonicated brains. (TIF) [file pone.0057682.s002.tif]

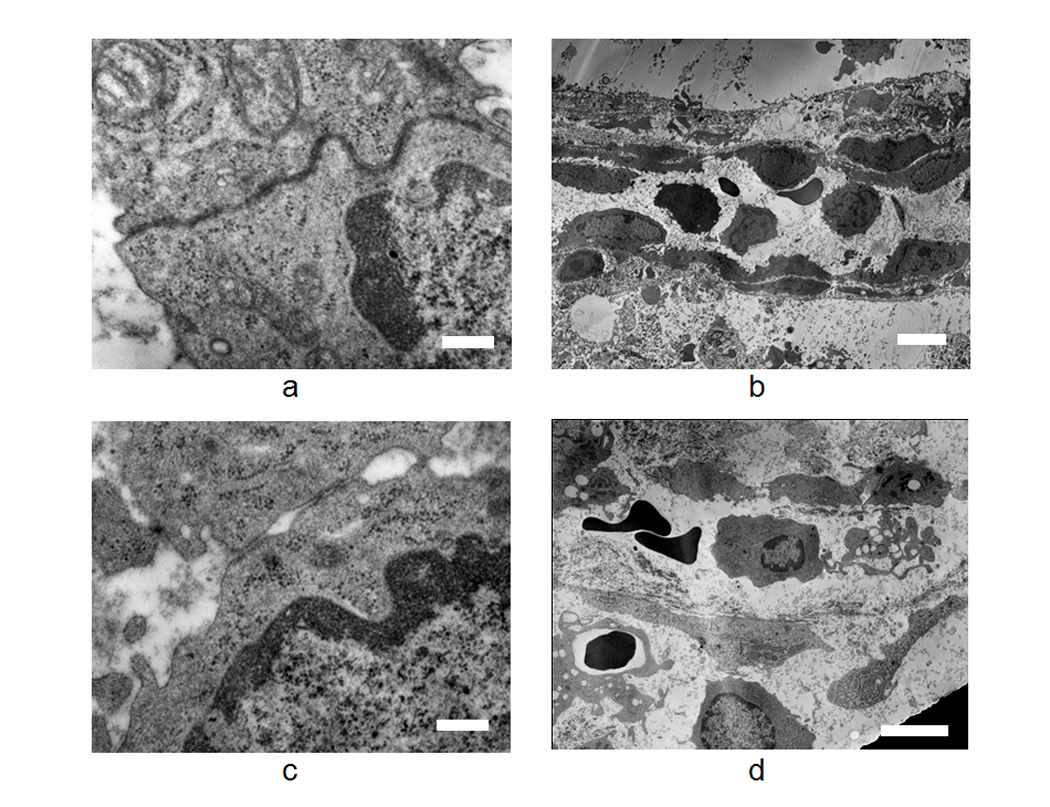

Supplement: Figure S3 — Transmission electron micrographs (TEMs) of control and FUS-exposed CNS capillaries. (a) Control capillary showing intact tight junction structure (bar = 115 nm). (b) Capillaries after 0.53-MPa FUS exposure revealing compromised tight junctions and numerous vesicles in endothelial cell cytoplasm (bar = 115 nm); (c) magnified tight junction from (b) showing inter-endothelial craft induced by FUS (bar = 1500 nm); (d) capillaries with 0.7-MPa FUS exposure showing large inter-endothelial tight-junction craft (bar = 115 nm). (TIF) [file pone.0057682.s003.tif]

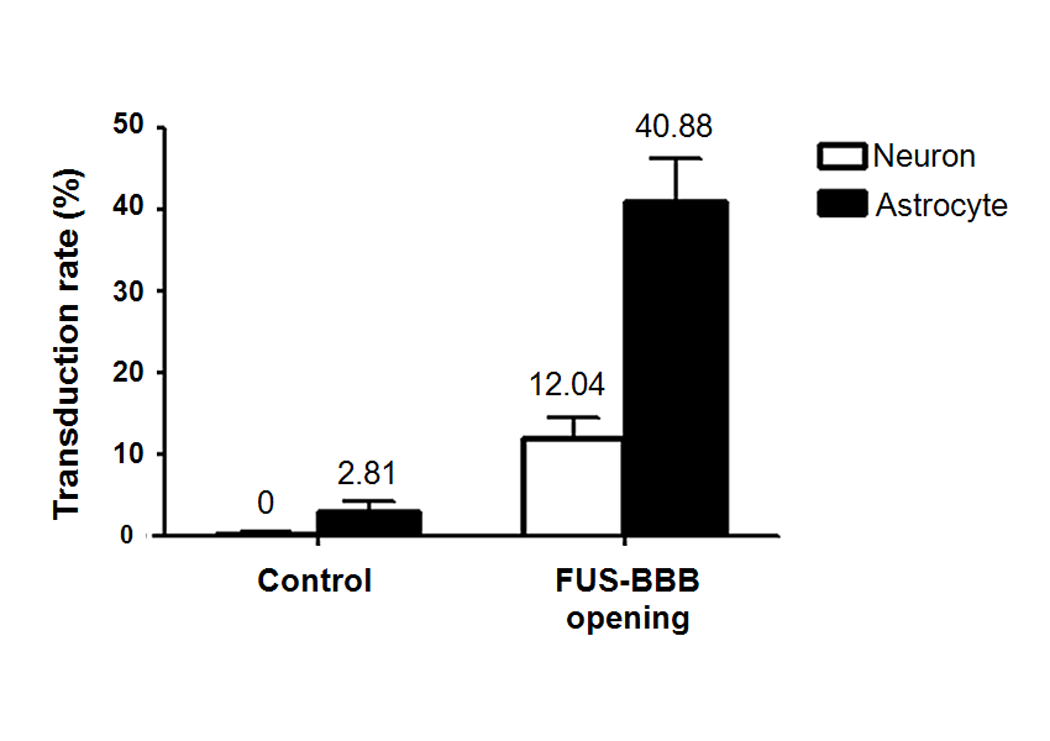

Supplement: Figure S4 — Cell-type specific AAV transduction. Here we show the comparison of AAV transduction rate in glial cells and neurons in control and FUS-BBB opened brain regions. (TIF) [file pone.0057682.s004.tif]

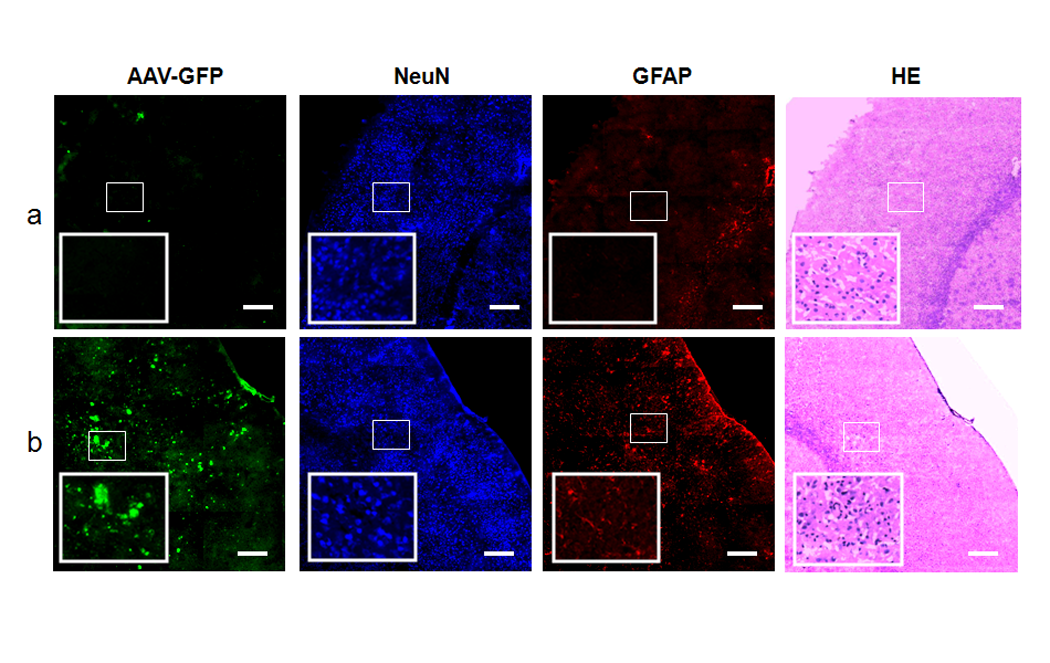

Supplement: Figure S5 — Immunofluorescence confirmation of AAV2-GFP expression. Neuronal Nuclei (NeuN) and Glial Fibrillary Acidic Protein (GFAP) immunofluorescence, and HE staining in (a) contralateral and (b) experimental brain. Neurons (nuclei stained by NeuN) appeared similar between the two sides of the brain, but glial cells were increased in the experimental lateral brain. HE-staining showed that the tissue structure was not severely damaged by FUS treatment. Bar = 200 µm. (TIF) [file pone.0057682.s005.tif]
