# Supplementary material for: Noninvasive and Targeted Gene Delivery into the Brain Using Microbubble-Facilitated Focused Ultrasound
Source: PLoS One. 2013 Feb 27;8(2):e57682. doi: 10.1371/journal.pone.0057682 (PMC3584045; doi:10.1371/journal.pone.0057682)
Supplement: Table S1 — Summary of numbers of animal used for focused ultrasound experiments. (DOCX) [file pone.0057682.s006.docx]

**Table S1.** Summary of numbers of animal used for focused ultrasound experiments (EB test = Evans blue BBB permeability evaluation test).

| **GFP observation time (Day)** | **EB**  **test** | **D7** | **D14** | **D21** | **D28** | **D35** | **D42** |  |
| --- | --- | --- | --- | --- | --- | --- | --- | --- |
| **FUS, 0.7MPa** | - | - | - | 2 | - | - | - |  |
| AAV-2 only | - | - | - | 2 | - | - | - |  |
| **FUS, 0.44MPa** | 2 | 1 | 1 | 3 | 1 | - | 4 |  |
| **FUS, 0.53MPa** | 2 | 2 | 4 | 4 | 3 | 4 | 8 |  |
| **FUS, 0.7MPa** | 2 | 3 | 6 | 12 | 4 | 3 | 4 |  |
| **Direct injection** | - | - | - | 5 | - | - | - | **Total** |
| **Sub-total** | 6 | 6 | 11 | 28 | 8 | 7 | 16 | **82** |
