# Supplementary material for: Noninvasive and Targeted Gene Delivery into the Brain Using Microbubble-Facilitated Focused Ultrasound
Source: PLoS One. 2013 Feb 27;8(2):e57682. doi: 10.1371/journal.pone.0057682 (PMC3584045; doi:10.1371/journal.pone.0057682)
Supplement: Method S1 — Cloning of AMCase and setup of AMCase-overexpressing cell line and rAAV. This supplemental methods section provides a detailed description of the AMCase cloning, the setup of AMCase-overexpressing cell line and the production of recombinant AAV. (DOCX) [file pone.0057682.s007.docx]

**Method S1. Cell culture, cloning of AMCase and setup of AMCase-overexpressing cell line and rAAV.**

3T3 and 293T cell lines were cultured and maintained in Dulbecco’s modified Eagle’s medium (DMEM; GIBCO Invitrogen, Carlsbad, CA) containing 10% fetal bovine serum (FBS; HyClone, Logan, UT) with 5% CO_2_ in humidified air at 37°C.

RNA was extracted from mouse lung tissues with TRIzol reagent (Invitrogen) and reverse-transcribed (RT) with a two-step RT-polymerase chain reaction (RT-PCR) kit (Invitrogen). The full-length AMCase sequence was amplified with the following primers: forward, 5’-ATCAGAATTCTATGGCCAAGCTACTTCTC-3’; and reverse, 5’-TTTCTGCGGCCGCATGGCATTAGGTTCATGGC-3’. The AMCase- overexpressing cell line was established by transfection of a pTriEx-neo vector carrying the AMCase sequence into 3T3 cells. A dilution series was carried out under G418 selection pressure.

Virus containing pAAV2-IRES-hrGFP was produced with the AAV2 helper system (Stratagene, La Jolla, CA). Briefly, plasmid DNA (pAAV2-IRES-hrGFP plasmid plus the pRC vector encoding Rep and Cap proteins and the pHelper vector encoding adenoviral gene products) was used to transfect 293T cells at 80% confluence. Cell lysates were collected 48 hr post-transfection and purified by CsCl density gradient centrifugation. Titers of rAAV-hrGFP were determined by RT-PCR analysis by calculating the viral genome copy number. The biodistribution of the transduced viral vector (pAAV2-IRES-hrGFP) is mainly in liver and spleen [[1-3](#_ENREF_1)], and transduction efficiency can be high in other organs when specific promoters are designed [[4](#_ENREF_4)]. No neurotoxicity is detected when the viral particle titer is below 10^11^ vg with an injection volume of 30 μL in mice [[5](#_ENREF_5),[6](#_ENREF_6)] .

**References:**

1. Hu C, Busuttil RW, Lipshutz GS (2010) RH10 provides superior transgene expression in mice when compared with natural AAV serotypes for neonatal gene therapy. J Gene Med 12: 766-778.

2. Ponnazhagan S, Mukherjee P, Yoder MC, Wang XS, Zhou SZ, et al. (1997) Adeno-associated virus 2-mediated gene transfer in vivo: organ-tropism and expression of transduced sequences in mice. Gene 190: 203-210.

3. Wu Z, Asokan A, Samulski RJ (2006) Adeno-associated virus serotypes: vector toolkit for human gene therapy. Mol Ther 14: 316-327.

4. Yang CJ, Liu YK, Liu CL, Shen CN, Kuo ML, et al. (2009) Inhibition of acidic mammalian chitinase by RNA interference suppresses ovalbumin-sensitized allergic asthma. Hum Gene Ther 20: 1597-1606.

5. Royo NC, Vandenberghe LH, Ma JY, Hauspurg A, Yu L, et al. (2008) Specific AAV serotypes stably transduce primary hippocampal and cortical cultures with high efficiency and low toxicity. Brain Res 1190: 15-22.

6. Howard DB, Powers K, Wang Y, Harvey BK (2008) Tropism and toxicity of adeno-associated viral vector serotypes 1, 2, 5, 6, 7, 8, and 9 in rat neurons and glia in vitro. Virology 372: 24-34.
