# Supplementary material for: Noninvasive and Targeted Gene Delivery into the Brain Using Microbubble-Facilitated Focused Ultrasound
Source: PLoS One. 2013 Feb 27;8(2):e57682. doi: 10.1371/journal.pone.0057682 (PMC3584045; doi:10.1371/journal.pone.0057682)
Supplement: Method S2 — Real-time PCR and Western blotting. This supplemental methods section provides a detailed description of the use of real-time PCR and Western blotting to confirm the GFP expression in the brain. (DOCX) [file pone.0057682.s008.docx]

**Method S2. Real-time PCR and Western blotting**

Expression of humanized *Renilla* green fluorescent protein (hrGFP), and actin was analyzed by real-time PCR, using a LightCycler PCR system (Roche, Indianapolis, IN). Actin 300 (forward primer, 5’-GAAACTACATTCAATTCCATC-3’; reverse primer, 5’-CTAGAAGCACTTGCGGTGCAC-3’) was used as a housekeeping control gene. The reaction parameters for actin 300 and hrGFP amplification (forward primer, 5’-ATGGTGAGCAAGCAGATCCTG-3’; reverse primer, 5’-GGTGCGCTCGTACACGAAGCC-3’) were as follows: initial denaturation at 95°C for 10 min, followed by 35 cycles of 95°C for 10 sec, 50°C for 10 sec, and 72°C for 10 sec.

In Western blotting, mice brain tissues were washed twice in ice-cold PBS and lysed on ice in ice-cold T-PER tissue protein extraction reagent (Pierce, Rockford, IL, USA) containing protease inhibitor cocktail (Sigma, St. Louis, MO, USA). Lysates were cleared by centrifugation, and total protein concentrations were determined by Bradford assay (Bio-Rad, Hercules, CA, USA). Protein samples were separated on 12% polyacrylamide gels by SDS–PAGE and transferred to polyvinylidene difluoride membranes (Millipore). Blots were blocked overnight in TBS (20 mM Tris-HCl, 150 mM NaCl, 0.1% Tween-20, 0.5 μM EDTA, pH 7.4) containing 5% nonfat dry milk, incubated for 2 h with antibodies (anti-GFP: 1:10,000, Abcam, Cambridge, UK; β-tubulin, 1:10,000, Sigma-Aldrich, MO, USA), and then incubated with horseradish peroxidase-conjugated goat anti-mouse IgG (1:20,000; PerkinElmer, MA, USA) for 1 h. Signals was detected using the Western Lightning kit (PerkinElmer, MA, USA) according to the manufacturer’s instructions.
