# Supplementary material for: Noninvasive and Targeted Gene Delivery into the Brain Using Microbubble-Facilitated Focused Ultrasound
Source: PLoS One. 2013 Feb 27;8(2):e57682. doi: 10.1371/journal.pone.0057682 (PMC3584045; doi:10.1371/journal.pone.0057682)
Supplement: Method S3 — Focused ultrasound calibration and assessment of blood-brain barrier disruption. This supplemental methods section provides a detailed description of focused ultrasound calibration and measurement, as well as the use of Evans Blue (EB) infiltration and staining to assess the BBB-opening. (DOCX) [file pone.0057682.s009.docx]

**Method S3. Focused ultrasound calibration and Assessment of blood-brain barrier disruption.**

A focused ultrasound transducer (Imasonics, Besancon, France; diameter: 60 mm, radius of curvature: 80 mm, frequency: 1.5 MHz, electric-to-acoustic efficiency: 35%) was used to generate a beam of ultrasound energy. A four-channel amplifier (Model 500-019, Advanced surgical systems, Inc., Tucson, AZ USA) was used to generate the driving signal and to monitor the power delivered to the transducer. The ultrasound pressure field was measured to characterize the focal region in an acrylic water tank with a semiautomatic 3D positioning system (with the consideration of mouse-skull insert loss; the mean pressure loss caused by the skull was measured to be 12%). A polyvinylidene fluoride (PVDF)-type hydrophone was used to measure the pressure distribution along the axis of the transducer and in the radial direction (Onda, Sunnyvale, CA; calibration range: 50 kHz to 20 MHz). The half-maximum pressure amplitude diameter and length of the produced focal spot were 2 and 10 mm, respectively. Pressure was measured at low hydrophone output amplitudes.

The impact of standing wave on transcranial focused ultrasound exposure has been discussed thoroughly in previous studies [[1](#_ENREF_1),[2](#_ENREF_2)]. O’Reilly et al. investigated standing-wave effects in rat skull and concluded that the scale of relative standing-wave fluctuation (RSWF) significantly affected by the ratio between cranial cavity and FUS wavelength [[1](#_ENREF_1)]. In our study, delivering 1.5 MHz exposure (wavelength = 1 mm) to mouse cranial cavity (about 10-12 mm) contributes to a ratio of 10-12. This ratio, which is analogous to the human skull test under 230-kHz FUS exposure reported in Song et al [[2](#_ENREF_2)], may contribute to a possible RSWF of ±12.5% in pressure amplitude. Increasing the driving frequency may directly reduce the standing wave effect, yet the skull penetration of ultrasonic wave will be greatly reduced. Possible solutions includes the use of reduced duty cycle [[1](#_ENREF_1)] or exposure using sharply focused transducer [[2](#_ENREF_2)] have been reported to have significant standing-wave elimination effect.

To access FUS-BBB opening, Evans Blue (EB) dye extravasations were used to evaluate BBB permeability produced by the varying FUS exposure pressures in six extra animals. The mice were injected IV with EB (Sigma, St. Louis, MO) at a concentration of 30 mg/kg immediately after FUS application and were sacrificed approximately 3 hours later. Mice were perfused with saline via the left ventricle followed by removal of the brains. Tissues were coronally sectioned into 3 slices from 3 mm anterior to 3 mm posterior to the bregma and then divided into control and experimental hemispheres before measuring the amount of EB extravasated. Brain hemispheres were separately weighed and soaked in formamide (1 ml/100 mg, Sigma, St. Louis, MO) at 60°C for 24 hours. The sample was centrifuged for 20 min at 14000 r.p.m. at 4°C. The concentration of dye extracted from each brain was determined by spectrophotometer (Multiskan FC, Thermo Scientific, CA, USA) at 620 nm and compared with a standard curve created by recording optical densities (O.D.) from serial dilutions of a known concentration of EB in 0.9% sodium chloride solution. The tissue content of EB was quantified by linear regression using the standard curve derived from seven concentrations of the dye and was denoted as the amount per gram of tissue. Differences among EB concentrations were analyzed with a t-test. Statistical significance was defined as p values＜0.05.

We also examined endothelial cell and tight-junctional changes after focused ultrasound exposures by using Transmission Electron Microscopy (TEM). Fresh tissues were fixed in phosphate buffer containing 30 mg/ml glutaraldehyde, postfixed in 10 mg/ml osmium tetroxide, dehydrated through a graded series of alcohol, and finally embedded in Epon 812 epoxy resin. Ultrathin sections (50 nm) were stained with uranyl acetate and lead citrate and examined using a Hitachi H-7500 transmission electron microscope.

**References:**

1. O'Reilly MA, Huang Y, Hynynen K (2010) The impact of standing wave effects on transcranial focused ultrasound disruption of the blood-brain barrier in a rat model. Phys Med Biol 55: 5251-5267.

2. Song J, Pulkkinen A, Huang Y, Hynynen K (2012) Investigation of standing-wave formation in a human skull for a clinical prototype of a large-aperture, transcranial MR-guided focused ultrasound (MRgFUS) phased array: an experimental and simulation study. IEEE Trans Biomed Eng 59: 435-444.
