# Supplementary material for: Noninvasive and Targeted Gene Delivery into the Brain Using Microbubble-Facilitated Focused Ultrasound
Source: PLoS One. 2013 Feb 27;8(2):e57682. doi: 10.1371/journal.pone.0057682 (PMC3584045; doi:10.1371/journal.pone.0057682)
Supplement: Method S4 — AAV direct injection as a positive control. This supplemental methods section provides a detailed description of the AAV direct injection as a positive control group. (DOCX) [file pone.0057682.s010.docx]

**Method S4. AAV direct injection as a positive control.**

Intracranial injections were performed with a rodent stereotaxic frame (Stoelting, Wood Dale, IL) and mice were anesthetized with a mixture of oxygen (flow rate: 0.8 L/min) and 2% vaporized isoflurane using an anesthesia vaporizer. Viral vectors were injected in the left midstriatum using the following coordinates from bregma in mm:(AP +0.5, ML +2.0, DV −2.5.). All infusions were performed using a microsyringe pump controller (SP-M2, DR instruments, Taiwan) attached to aHamilton syringe with a 30-gauge needle (Hamilton Co., Rena, NV). Viral vector was injected into the striatum in an accepted volume of 3 μl (3 x 10^9^ genome copies) at a rate of 200 nl/min. After injection, the needle was left in place for 5 min before slow withdrawal.
